# Population Dynamics of *Lactobacillus helveticus* in Swiss Gruyère-Type Cheese Manufactured With Natural Whey Cultures

**DOI:** 10.3389/fmicb.2018.00637

**Published:** 2018-04-04

**Authors:** Aline Moser, Karl Schafroth, Leo Meile, Lotti Egger, René Badertscher, Stefan Irmler

**Affiliations:** ^1^Agroscope, Bern, Switzerland; ^2^Laboratory of Food Biotechnology, Institute of Food, Nutrition and Health, ETH Zürich, Zurich, Switzerland

**Keywords:** *Lactobacillus helveticus*, population dynamics, strain diversity, cheese ripening, natural whey cultures

## Abstract

*Lactobacillus helveticus*, a ubiquitous bacterial species in natural whey cultures (NWCs) used for Swiss Gruyère cheese production, is considered to have crucial functions for cheese ripening such as enhancing proteolysis. We tracked the diversity and abundance of *L. helveticus* strains during 6 months of ripening in eight Swiss Gruyère-type cheeses using a culture-independent typing method. The study showed that the *L. helveticus* population present in NWCs persisted in cheese and demonstrated a stable multi-strain coexistence during cheese ripening. With regard to proteolysis, one of the eight *L. helveticus* populations exhibited less protein degradation during ripening.

## Introduction

Natural whey cultures (NWCs) are undefined cheese-starter cultures that are obtained by back-slopping techniques. The whey from the previous cheesemaking process is kept, incubated at a defined temperature for a certain length of time, and then added again to milk to initiate milk acidification. NWCs are used in Italy for the manufacture of Grana Padano, Parmigiano Reggiano, Mozzarella di Bufala Campana, Provolone Valpadane, and Pecorino Romano cheese, and in Switzerland for Gruyère PDO cheese (Gatti et al., 2014). The microbial composition of NWCs used for the manufacture of Grana Padano and Parmigiano Reggiano have been well studied and it was found that *Lactobacillus helveticus, Streptococcus thermophilus*, and *Lactobacillus delbrueckii* subsp. *lactis* form the core microbiome of these cultures (Gatti et al., 2014). On the contrary, little is known about the microbial composition of NWCs used for the manufacture of Swiss Gruyère PDO cheese. The procedure of manufacture is described in the specifications of Gruyère PDO (https://gruyere.com/en/specifications/) of which some important points are briefly outlined below: Gruyère PDO is produced from raw cow's milk which must be transformed in open copper vats. The use of in-house NWCs is obligatory and they are usually added in the per mille range. Coagulation is done with the aid of rennet at 31°C and lasts between 30 and 50 min. Heating of the curd is performed in a temperature range between 54 and 59°C. The maturation takes place between 12 and 18°C and a relative humidity of approximately 92% for at least 5 months.

We have previously found that *L. helveticus* is a predominant species in those NWCs (Moser et al., 2017a). A metagenomics study performed on Gruyère PDO NWCs further revealed the presence of *S. thermophilus* and *L. delbrueckii* subsp. *lactis* (Schmid et al., 2018).

*Lactobacillus helveticus* is a nutritional fastidious bacterium that has multiple amino acid auxotrophies (Hebert et al., 2000; Christensen and Steele, 2003). To overcome these auxotrophies *L. helveticus* possesses a large protease and peptidase complement that liberates amino acids through the breakdown of proteins and peptides (Slattery et al., 2010; Griffiths and Tellez, 2013). The proteolytic system of *L. helveticus* has been associated with biotechnologically important traits, such as faster cheese ripening and enhanced flavor development (Slattery et al., 2010). Due to the liberation of bioactive peptides, which are associated with health-promoting effects, *L. helveticus* is increasingly studied with regard to it being a probiotic species (Taverniti and Guglielmetti, 2012).

In Emmental cheese the presence of *L. helveticus* has been associated with late fermentation (Fröhlich-Wyder and Bachmann, 2004). Late fermentation is a defect induced by excessive proteolysis and carbon dioxide production, which finally leads to undesired openings such as splits and cracks, as well as reduced elasticity. The appearance of splits and cracks in Gruyère PDO cheese leads to a downgrading of the cheese. Based on the connection of *L. helveticus* with late fermentation in Emmental cheese, Gruyère cheesemakers assume that undesired openings in Gruyère PDO cheese are related to the presence of *L. helveticus* strains. However, this hypothesis has never been investigated using scientific methods. We have recently established a high-throughput amplicon sequencing method to profile *L. helveticus* populations in dairy products (Moser et al., 2017b). In essence, the method determines the single nucleotide polymorphisms present in regions of the *L. helveticus slpH* gene, which encodes a surface-layer protein. During data analysis, the identified genetic variations are assigned to sequence types (STs). By using this approach, we found that various *L. helveticus* STs co-occur in NWCs collected from the Gruyère region. The method can be applied to monitor *L. helveticus* strain diversity in cheese and relate it to cheese quality.

In the present study, we found that the *L. helveticus* ST diversity remained during cheese ripening. Furthermore, we observed a potential functional relevance of *L. helveticus* populations for protein degradation in cheese.

## Materials and methods

### NWC collection and cheesemaking

Eight different NWCs (A–H) were collected at eight geographically separated cheese factories that produce Swiss Gruyère PDO cheese. These NWCs were obtained by the incubation of the whey from the cheese production of the previous day at 38°C for 20 h. The NWCs were transported to the Agroscope pilot plant using ice packages and then immediately used as starters for the production of Swiss Gruyère-type cheeses. Eight model cheeses were produced in stainless-steel vats from 90 L of cow's evening milk that had been collected from a Gruyère cheesemaking factory and stored over night at 5°C. For cheesemaking, the milk was warmed at 31°C and 1 L of CuSO_4_ solution (0.025%, w/v) was added to simulate the leaching of copper ions—a process that takes place when copper vats are utilized for milk tranformation. Then, 90 mL of NWC were added as the only starter culture. After incubation at 31–32°C for 30 min, the coagulation of milk was performed by the addition of 13 mL of calf rennet (Winkler GR orange, Winkler AG, Konolfingen, Switzerland; approximately 194 International Milk Clotting Units mL^−1^) followed by incubation at 32°C for 35 min. The coagulum was cut into 3–6 mm diameter granules, and the curd was heated to 56°C over 40 min. The curd was molded and pressed at 6 bar over 4 h. The temperature in the pressing chamber was lowered from 55 to 25°C over 20 h. The cheeses were salted in brine at 11–13°C for 14 h and ripened at 14–15 and 90–96% relative humidity. Samples were taken after 24 h, 1, 2, 3, and 6 months of ripening.

### Chemical analysis

Total nitrogen (TN), water-soluble nitrogen (WSN), and 12% trichloroacetic acid-soluble nitrogen (NPN) were analyzed as described by Collomb et al. (1990). The pH-value of the cheeses was determined using a pH electrode (Knick Elektronische Messgeräte GmbH & Co. KG, Berlin, Germany). For the determination of lactate and galactose, 1.25 g of cheese were homogenized in 50 mL of water using an OmniPrep Multi-Sample Homogenizer (Omni International, Kennesaw, USA). For the determination of citrate, 5 g of cheese were used. The homogenates were then incubated at 2°C for 20 min. Particles and fat were removed by filtration. The concentration of D- and L-lactate, galactose, and citrate in the filtrates was determined using commercial enzymatic assay kits (R-Biopharm AG, Murten, Switzerland). The amount of total free amino acids in the cheese extracts was estimated using the o-phthalaldehyde (OPA) method as described by Kopf-Bolanz et al. (2012).

### Microbiological analysis

Cheese samples were homogenized 1:10 in peptone water at 40°C (10 g L^−1^ peptone from casein, 5 g L^−1^ sodium chloride, 20 g L^−1^ trisodium citrate dihydrate, pH 7.0) using a stomacher (Masticator, IUL Instruments, Königswinter, Germany). The homogenate was then serially diluted in NA (8 g L^−1^ sodium chloride, 1 g L^−1^ peptone from caseine [pH 7.2]) and plated on agar plates containing MRS (De Man et al., 1960), M17 (Terzaghi and Sandine, 1975) containing 5 g L^−1^ D-glucose, and FH (Isolini et al., 1990). The latter medium contains mannitol as the main source of energy and vancomycin and is used to cultivate and enumerate facultatively heterofermentative lactic acid bacteria (FHL). Three replicates were performed for each sample. The MRS agar plates were incubated at 37°C for 72 h, M17-glucose agar plates at 37°C for 48 h, and FH agar plates at 30°C for 72 h. The agar plates were incubated under anaerobic conditions.

### Estimation of *L. helveticus* using quantitative PCR

Genomic DNA (gDNA) from NWC and cheese samples was extracted as described by Moser et al. (2017a). Prior to gDNA extraction all bacterial pellets were treated with TURBO^TM^ DNase (TURBO DNA-free Kit, Thermo Fisher Scientific, Reinach, Switzerland) to remove DNA from lysed cells. The presence and abundance of *L. helveticus* in the samples was determined by quantitative PCR (qPCR), which specifically targets the *pheS* gene encoding the alpha-subunit of the phenylalanine-tRNA synthetase (Moser et al., 2017a). Samples containing *L. helveticus* were further subjected to *L. helveticus* population profiling.

### *Lactobacillus helveticus* population profiling

To determine *L. helveticus* populations, the culture-independent method described by Moser et al. (2017b) was performed. Briefly, an approximately 1,000-bp region of the *slpH* gene was amplified by PCR and the derived amplicons were fragmented by sonication. The fragments were barcoded using the Ion Xpress Barcode Adapter 1–16 kit (Thermo Fisher Scientific), and the barcoded libraries were sequenced on an Ion 316^TM^ chip and the Ion PGM Hi-Q View Sequencing kit on an Ion Torrent sequencer (Thermo Fisher Scientific). On each chip, 16 barcoded libraries were sequenced.

## Results

### Chemical analyses

The pH and the formation of D-lactate and L-lactate in cheese was measured after 24 h of ripening. The pH was on average at 5.1, and the total lactate concentrations in the eight cheeses were between 153 and 159 mmol kg^−1^ cheese (Table 1). The L-lactate/D-lactate ratio was similar in the different cheeses with the exception of cheese F, which showed a high L-/D-lactate ratio of 5.2. During ripening the level of the total amount of lactate decreased to an average of 94 mmol kg^−1^ cheese. The unusual L-/D-lactate ratio remained high in the 6-month-old cheese F (ratio of 4.2, Table 1). The level of citrate, which is an indicator for the presence of citrate-fermenting mesophilic bacteria, was above 4.9 mmol kg^−1^ in all cheeses after 6 months of ripening.

**Table 1:** Chemical properties of the eight cheeses (A–H) during ripening.

	**A**	**B**	**C**	**D**	**E**	**F**	**G**	**H**
**AFTER 24 H**								
TL (mmol kg^−1^)	158	158	153	155	157	155	155	159
LLA (mmol kg^−1^)	98	107	83	74	88	130	69	86
DLA (mmol kg^−1^)	60	51	70	81	69	25	86	73
LLA/DLA ratio	1.63	2.10	1.18	0.91	1.28	5.2	0.80	1.18
Gal (mmol kg^−1^)	0.3	0.4	1.25	0	0.3	0.6	0	0
pH	5.11	5.13	5.10	5.13	5.13	5.13	5.14	5.09
**AFTER 6 MONTHS**								
TL (mmol kg^−1^)	87	91	87	92	94	100	98	103
LLA (mmol kg^−1^)	50	56	47	41	48	81	40	53
DLA (mmol kg^−1^)	37	35	40	51	46	19	58	50
LLA/DLA ratio	1.35	1.60	1.18	0.80	1.04	4.26	0.69	1.06
FAA (mmol kg^−1^)	380	385	375	357	380	382	374	321
CA (mmol kg^−1^)	5.4	5.4	6.3	6.6	4.7	4.9	5.0	6.9
TN (g kg^−1^)	41.09	41.57	39.73	42.69	40.68	41.49	40.80	40.61
NPN (g kg^−1^)	9.64	9.38	9.11	9.37	9.96	9.80	9.44	8.41
WSN (g kg^−1^)	13.09	12.64	12.41	13.27	13.17	13.39	13.07	12.57

*TL, total lactate; LLA, L-lactate; DLA, D-lactate; Gal, galactose; FAA, total free amino acids; CA, citrate; TN, total nitrogen; NPN, non-protein nitrogen; WSN, water-soluble nitrogen*.

To evaluate the proteolysis in the cheese after 6 months of ripening, TN and fractions containing the WSN, acid-soluble nitrogen, and free amino acids were determined (Table 1). We found that the cheese produced with NWC H in comparison to the other cheeses exhibited considerably lower levels of non-protein nitrogen (8.41 g kg^−1^) and free amino acids (312 mmol kg^−1^).

### Microbiological analyses

Lactobacilli and streptococci were enumerated using MRS and M17-glucose agars, respectively. The cultivable lactobacilli and streptococci reached a minimum log_10_ value of 6.81 and 7.09 in 24-h old cheese (Table 2). Remarkably, we did not observe colonies on MRS agar for the cheese manufactured with NWC A. Lactobacilli were detected and counted in the afore-mentioned cheese after 4 weeks of ripening (Table 2).

**Table 2:** Population densities determined in cheese at different time points with different agar media.

**Time**	**Bacterial count (log_10_ CFU g^−1^ cheese)**				
	**MRS^a^ (Lactobacilli)**	***p*-value^b^**	**M17x^a^ (Streptococci)**	***p*-value^b^**	**FH^a^ (FHL)**
**CHEESE A**					
24 h	–	–	7.85 ± 0.08	–	–
1 month	4.46 ± 0.12	–	6.03 ± 0.09	0.0014	–
2 months	4.41 ± 0.08	0.665	4.5 ± 0.06	0.0022	–
3 months	4.83 ± 0.08	0.0543	4.64 ± 0.03	0.0244	–
6 months	3.3	–	2.56 ± 0.07	0.0005	–
**CHEESE B**					
24 h	6.23 ± 0.15	–	7.46 ± 0.04	–	–
1 month	6.88 ± 0.03	0.0402	6.66 ± 0.07	0.0012	–
2 months	6.37 ± 0.02	0.0128	5.89 ± 0.09	0.0035	–
3 months	5.9 ± 0.02	0.4827	5.96 ± 0.02	0.7814	–
6 months	3.3	–	3.27 ± 0.05	< 0.0001	–
**CHEESE C**					
24 h	8.77 ± 0.02	–	8.61 ± 0.02	–	–
1 month	7.32 ± 0.05	0.0257	7.26 ± 0.02	0.0009	4.32 ± 0.05
2 months	7.86 ± 0.02	0.0112	4.5 ± 0.06	0.0003	5.14 ± 0.03
3 months	5.21 ± 0.04	0.0040	5.0 ± 0.01	0.0047	–
6 months	5.87 ± 0.04	0.0028	5.76 ± 0.03	0.0007	5.81 ± 0.1
**CHEESE D**					
24 h	8.45 ± 0.02	–	9.02 ± 0.02	–	–
1 month	7.41 ± 0.11	0.0037	7.43 ± 0.02	< 0.0001	–
2 months	7.4 ± 0.03	0.2589	7.08 ± 0.02	0.0020	–
3 months	7.45 ± 0.04	0.4907	7.28 ± 0.01	0.0020	–
6 months	5.78 ± 0.05	0.0003	5.5 ± 0.04	0.0002	5.4 ± 0.02
**CHEESE E**					
24 h	7.25 ± 0.08	–	8.17 ± 0.05	–	–
1 month	6.03 ± 0.03	0.0084	6.17 ± 0.17	0.0018	–
2 months	5.4 ± 0.02	0.0054	4.95 ± 0.09	0.0100	–
3 months	4.2 ± 0.06	0.0171	6.56 ± 0.06	0.0005	–
6 months	3	–	4.57	–	–
**CHEESE F**					
24 h	6.81 ± 0.08	–	7.22 ± 0.03	–	–
1 month	7.06 ± 0.03	0.0901	7.13 ± 0.05	0.7135	5.1 ± 0.04
2 months	7.25 ± 0.15	0.2651	7.08 ± 0.05	0.5525	5.31 ± 0.03
3 months	7.03 ± 0.09	0.9484	7.14 ± 0.07	0.4223	–
6 months	4.47 ± 0.08	0.0005	4.2 ± 0.05	0.0014	–
**CHEESE G**					
24 h	7.20 ± 0.15	–	8.52 ± 0.03	–	–
1 month	6.5 ± 0.03	0.0581	6.77 ± 0.02	< 0.0001	–
2 months	6.68 ± 0.01	0.2925	6.12 ± 0.23	0.051	–
3 months	6.09 ± 0.06	0.0007	5.08 ± 0.15	0.1017	–
6 months	4.22 ± 0.16	0.0037	3.63 ± 0.17	0.1287	–
**CHEESE H**					
24 h	7.36 ± 0.07	–	7.09 ± 0.13	–	–
1 month	8.0 ± 0.04	0.0637	7.43 ± 0.01	0.0342	–
2 months	7.08 ± 0.03	0.2977	6.89 ± 0.02	0.8477	–
3 months	6.03 ± 0.06	0.0026	5.68 ± 0.02	0.0005	–
6 months	7.86 ± 0.02	0.0005	6.42 ± 0.01	0.0747	5.75 ± 0.05

a *Colony forming units (CFU): numbers represent the log_10_ mean and the log_10_ standard deviation*.

b *p-values were obtained by comparing the mean CFU values at a given sampling time point with the mean values of the preceding time point with a paired t-test in R (version 3.3.2)*.

Moreover, the numbers of counted colonies declined during ripening. On MRS agar plates, the bacterial count declined from 7.44 (±0.89) log_10_ CFU g^−1^ cheese (mean value from all cheese productions) after 24 h of ripening to 4.73 (±1.67) log_10_ CFU g^−1^ cheese after 6 months of ripening with a mean decline of 2.51 log_10_ CFU g^−1^ cheese between 24 h and 6 months of ripening (Table 2). On M17-glucose agar plates, the mean decline between 24 h and 6 months of ripening was 3.5 log_10_ CFU g^−1^ cheese, with 7.99 (±0.7) log_10_ CFU g^−1^ cheese after 24 h of ripening, and 4.49 log_10_ CFU g^−1^ cheese after 6 months of ripening (Table 2).

To detect non-starter lactic acid bacteria, typically facultatively heterofermentative lactobacilli and pediococci, we used FH agar. Colonies were found after 1 and 2 months of ripening in cheese C and in cheese F, but not after 3 months of ripening. After 6 months of ripening, bacterial growth on the FH agar plates was detected for cheeses C, D, and G (Table 2).

### Enumeration of *L. helveticus* in NWC and cheese

**Figure 1:**
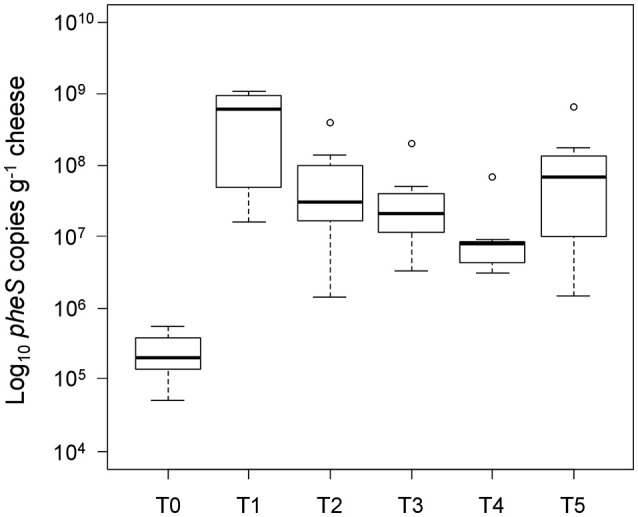
Quantification of *L. helveticus* during cheese ripening. The number of intact *L. helveticus* cells was monitored by qPCR using DNA from the whole-cell fractions. Samples were taken after 24 h (T1), 1 month (T2), 2 months (T3), 3 months (T4), and 6 months (T5) of cheese ripening. The *L. helveticus* concentration in the vat milk after the addition of the NWC (T0) was calculated from the concentration measured in the NWC. The boxplots show the median values from the cheese productions (*n* = 8).

We detected *L. helveticus* in all of the tested whole-cell fractions from NWC and cheese samples by qPCR. The *L. helveticus* concentration in the vat milk enriched with NWC was on average 5.38 (±0.35) log_10_ cells g^−1^ (T0 in Figure 1), assuming a single copy of *pheS* per cell. After 24 h of ripening the log_10_ *pheS* copy number g^−1^ increased to 8.54 (±0.75) (T1 in Figure 1). The number decreased to 7.5 (±0.72) log_10_ copies g^−1^ cheese after 1 month of ripening, 7.32(±0.56) log_10_ copies g^−1^ after 2 months of ripening, 6.92 (±0.4) log_10_ copies g^−1^ after 3 months of ripening, and was at 7.59 (±0.86) log_10_ copies g^−1^ at 6 months of ripening. A Wilcoxon rank sum test showed significant differences between the sample taken at T0 and all of the samples taken at the following five time points under a significance level of α = 0.01: *p* = 0.0002 for T0 and T1, *p* = 0.0002 for T0 and T2, *p* = 0.008 for T0 and T3, *p* = 0.008 for T0 and T4, and *p* = 0.008 for T0 and T5. The difference between samples at 24 h and 1 month was significant (*p* = 0.008) in contrast to non-significant differences (*p* = 0.51) between samples taken at 1 and 2 months of ripening. The *p*-value for the difference between samples taken at 2 and 3 months as well as at 3 and 6 months of ripening was significant with *p*-values of 0.008 and 0.04, respectively. An *L. helveticus* specific qPCR analysis did not detect a signal in cheese homogenates that had been filtrated through a sterile filter with a pore size of 0.22 μm (data not shown).

### *L. helveticus* diversity and abundance

**Figure 2:**
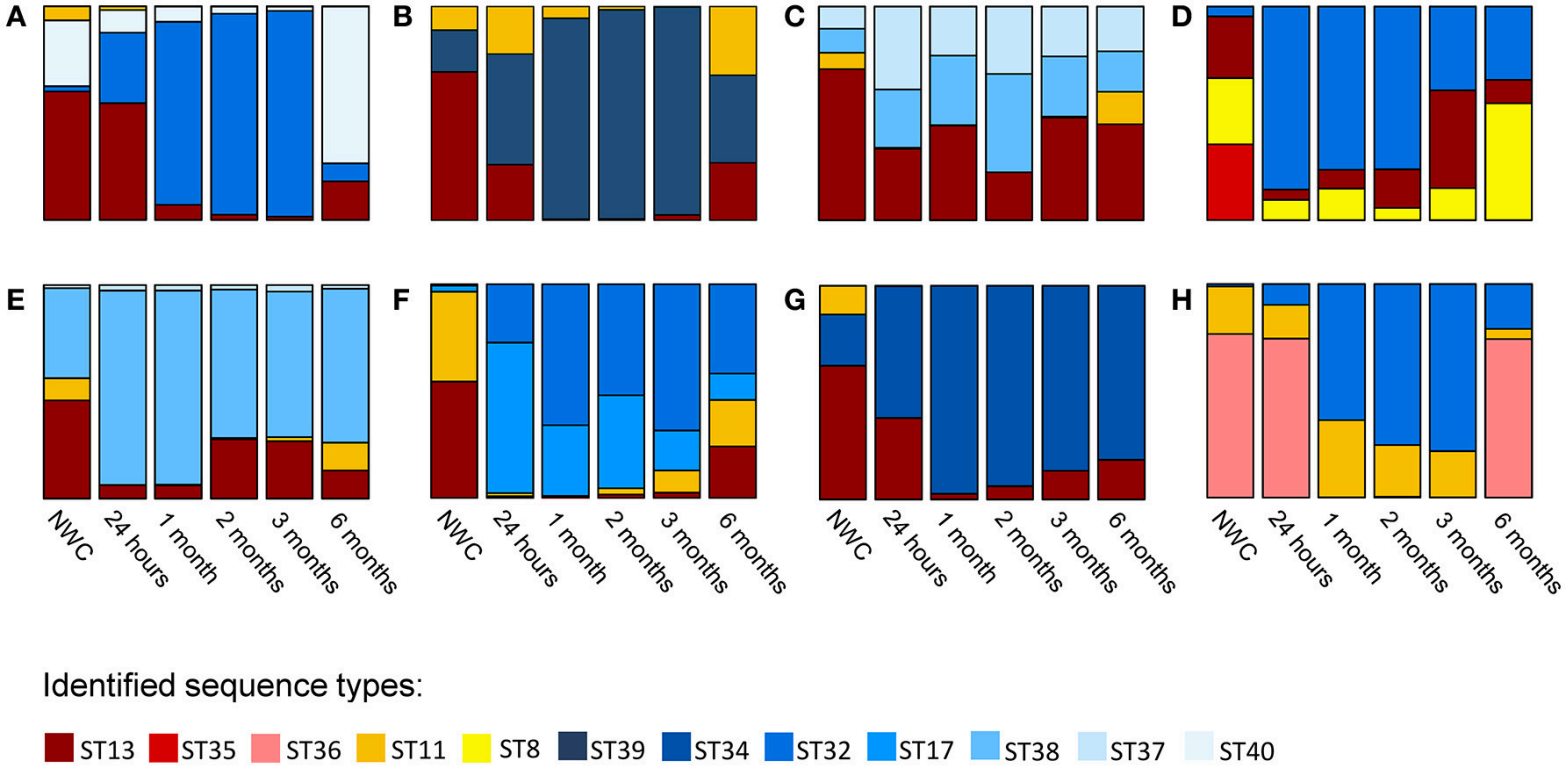
*Lactobacillus helveticus* strain composition in eight natural whey cultures (NWCs) **(A–H)** and in the corresponding eight cheeses during ripening. The *L. helveticus* strain composition was assessed for the eight NWCs and in the corresponding cheeses at five different time points during ripening. A specific color was assigned to each ST identified. The height of the different colors in the stacked bar plots represents the relative abundance (%) of each ST.

When we assessed the diversity of *L. helveticus* using high-throughput *slpH* amplicon sequencing, we detected a total of 12 different STs (indicated in 12 colors in Figure 2) in the eight cheese productions using NWCs from eight different cheese factories. The number of different STs per cheese production ranged from three to four. With two exceptions we found all STs present in NWCs in cheese after 6 months of ripening. The exceptions were ST35 of NWC D, which was not detected in any cheese sample, and ST11 of NWC G, which was not detected in the 6-month cheese. The relative abundance of STs fluctuated considerably during cheese ripening (Figure 2).

## Discussion

*Lactobacillus helveticus* is recognized as having a great importance for food fermentation and health-promoting properties (Giraffa, 2014). To exploit the potential of this species it is of interest to seek and characterize new interesting strains that can be applied in fermented food products. Besides the identification of new strains, fermented foods such as cheese are accessible and simple ecosystems that can be used as model systems to study the diversity and evolution of microbial communities (Wolfe and Dutton, 2015).

In the present study we were interested in estimating the potential of NWCs to find new strains and relating *L. helveticus* diversity with cheese proteolysis. Indeed, we detected seven new strains based on the polymorphisms present in the *slpH* locus. In all the cheeses, we found DNA from whole cells of *L. helveticus* during the entire ripening time. This result is in agreement with the findings of Gatti et al. (2008), who also detected whole cells from *L. helveticus* during the entire ripening time of 24-month-old Parmigiano-Reggiano cheeses, suggesting that intact cells of *L. helveticus* persist throughout the entire cheese ripening process.

Frequently, FHL grow in cheese made from raw milk. To evaluate the influence of FHL on the outcome of the study, we considered several aspects. First, the same batch of milk was used for the manufacture of the eight cheeses to ensure a consistent raw milk microbiome. Second, *Lactobacillus casei* and *Lactobacillus rhamnosus* are the dominant FHL present in Gruyère PDO cheese and can be found at population densities between 10^6^ and 10^7^ colony-forming units per gram of cheese (Casey et al., 2006). Therefore, we monitored FHL during cheese ripening using FH agar plates and found that colonies occured only occasionally and if at all with lower population densities in the cheeses than reported for Gruyère PDO (Table 2). Third, various chemical analysis were performed. Most cheeses revealed a similar profile for the measured parameters (Table 1). Of note was the observation that cheese F exhibited a high L-to-D lactate ratio after 24 h. It is known that *S. thermophilus* catabolizes carbohydrates solely into L-lactate, whereas *Lactobacillus* ssp. produce D- or DL-lactate (Parente and Cogan, 2004). Therefore, the high L-to-D lactate ratio in cheese F could be explained by a dominance of *S. thermophilus*. However, the equal population densities determined on M17-glucose agar (streptococci) and MRS agar (lactobacilli) do not support this hypothesis. Another explanation could be the presence of *Lactobacillus* ssp that have compromised D-lactate dehydrogenase activities. FHL can favor the racemization of L-lactate and we had previously observed lactate racemization in model cheeses in the presence of *L. casei* (Bogicevic et al., 2013). Therefore, we expected racemization of L-lactate in cheese F during ripening. However, a considerable decrease of the L-to-D ratio in cheese F was not observed after 6 month of ripening (Table 1) although FHL were detected after 2 and 3 months of ripening. In summary, we think that in the present study the contribution of FHL to cheese ripening and starter activities was marginal.

With regard to proteolysis we found a low value of free amino acids in cheese H when compared to the other cheeses (Table 1). This could probably be attributed to a lower bacterial peptidolytic activity in this cheese. Interestingly, cheese H is the only cheese that did not contain ST13 in its *L. helveticus* population. It is possible that this ST contributes to the high proteolytic activity observed in cheeses A–G. The isolation of ST13 and analysis of the proteolytic activity of this ST could reveal further insights into the functional diversity of *L. helveticus* populations. We cannot rule out that the phylogentically closely related *L. delbrueckii* subsp. *lactis* which possess also a considerable peptidase complement (Liu et al., 2010) also contributed to proteolysis. However, to better understand the role of this species in cheese ripening we need the development of typing methods and further investigation.

When we analyzed the strain composition of *L. helveticus* during ripening we detected the coexistence of several STs in all cheeses at all time points. The coexistence of strains of the same species in bacterial populations has been reported for different cheese varieties, and several next generation sequencing-based studies of samples originating from various habitats give evidence for the occurrence of co-existing intra-species diversity (Berthier et al., 2001; Gatti et al., 2003; Acinas et al., 2004; Rossetti et al., 2008; Caro-Quintero and Ochman, 2015). Unlike this previous research, our study followed the *L. helveticus* population over a time period of 6 months and thus provides insights into the dynamics of the observed diversity. We found major fluctuations in ST abundance in some samples, but in only one case did an ST disappear during the ripening time. Cheese is a challenging environment being scarce in nutrients and having a low pH and high osmolarity. Under such stressful conditions we expected competition to take place, which eventually leads to the extinction of all but one strain according to the “competitive exclusion principle,” a widely accepted maxim in community ecology (Hardin, 1960). However, the observed coexistence of multiple STs within cheese contradicted this assumption. Several explanations for the stable coexistence of intra-species diversity have been discussed in Ellegaard and Engel (2016). One explanation is that the different strains occupy different niches. Such micro-niches could be spatial, functional, or temporal (Ellegaard and Engel, 2016). The cheese matrix provides spatial niches. During the development of the casein matrix in the early stage of cheese production, bacteria become immobilized. This leads to a random and spatial distribution of bacterial colonies in the cheese matrix (Hickey et al., 2015). It is possible that the spatial separation between neighboring strains of the same species allows them to access all substrates necessary for growth subsequently avoiding competition. Competition between the different strains of a population can also be avoided if the strains specialize toward different functional niches. Due to biochemical changes during cheese ripening, the bacteria experience changes in the nutritional environment. Thus, for example, the pH rises due to the release of ammonia during proteolysis, which in turn results in the temporal variation of strain abundance (Ellegaard and Engel, 2016). Indeed, we observed major fluctuations in the relative abundance of STs at the different sampling time points. Such cyclic dynamics could also be explained by the presence of non-transitive competition (Laird, 2014). This mechanism describes a situation where a strain A outcompetes a strain B, which outcompetes a strain C, which, in turn, outcompetes strain A (A → B → C → A). In this non-transitive loop no strain is inferior or superior to the other strains at the population level (Kerr et al., 2002). Another explanation for fluctuations in strain composition over time could be the presence of bacteriophages. It was found that various strains of *Lactococcus lactis* and *Leuconostoc mesenteroides* coexisted in an undefined mesophilic starter culture that was propagated by back-slopping (Erkus et al., 2013). The authors also detected lytic phages in the supernatant of the starter culture and prophages in isolated strains and proposed that the presence of these phages stabilized the strain diversity during propagation because of a population density dependent phage predation (Erkus et al., 2013). In line with this study, we observed several incidents where the dominating ST was subjected to a substantial decrease between two sampling time points, such as ST32 between 3 and 6 months of ripening, ST36 between 24 h and 1 month of ripening, and ST39 between 3 and 6 months of ripening. The presence of bacteriophages in the dairy industry is well-known, and numerous *L. helveticus* phages have been isolated from NWCs without acidification problems in the current cheese production system (Zago et al., 2008). Additionally, it was shown that complete prophages reside within *L. helveticus* genomes (Schmid et al., 2018). Thus, we assume that *L. helveticus* bacteriophages play an important role in the microbial populations of the cheese environment.

The stable coexistence of several strains in the same ecological niche can also been explained by the “Black Queen Hypothesis” (BQH) (Morris et al., 2012). According to the BQH, the loss of a costly gene function provides a selective advantage to an individual if that function is leaky and can be provided by other members of the population. The loss of complementary genes within a population can lead to a network of interdependencies between the population members, which provides the conditions that favor a stable coexistence. In fact, sequencing and analysis of *L. helveticus* genomes provide evidence for reductive genome evolution in this species (Schmid et al., 2018) and support the hypothesis that the loss of gene functions is compensated by metabolic cooperation within *L. helveticus* populations.

## Conclusions

In the present study we showed the stability of *L. helveticus* diversity during cheese ripening. To our knowledge, this is the first study that has analyzed the population dynamics of *L. helveticus* during cheese ripening at the strain level. The stable coexistence of strains from the same species opens many questions that require future studies. It would be interesting to isolate the newly identified STs for further analyses. Linking genomic and phenotypic data could shed light on the micro-niche adaptations of specific strains or on possible inter-dependencies between different strains resulting from the deletion of complementary metabolic genes (Ellegaard and Engel, 2016).

Overall we can say that cheese is a valid model system for studying the nature of interactions in microbial communities. The application of culture-independent typing methods for *S. thermophilus* and *L. delbrueckii* subsp. *lactis* in the future will be valuable tools to expand our knowledge about the dynamics and interactions of microbial communities at the inter- and intra-species levels.

## Author contributions

AM, LM, and SI designed the study and wrote the manuscript. AM and SI also performed the microbiological analysis. Furthermore, AM performed the qPCR analyses and amplicon-based sequencing. KS performed the cheesemaking, ripening, and sampling. RB and LE were responsible for the chemical analyses.

### Conflict of interest statement

The authors declare that the research was conducted in the absence of any commercial or financial relationships that could be construed as a potential conflict of interest.
